# Occupational Safety and Health Conditions Aboard Small- and Medium-Size Fishing Vessels: Differences among Age Groups

**DOI:** 10.3390/ijerph14030229

**Published:** 2017-02-24

**Authors:** Mohamed A. Zytoon, Abdulrahman M. Basahel

**Affiliations:** 1 Department of Industrial Engineering, King Abdulaziz University, P.O. Box 80204, Jeddah 21589, Saudi Arabia; ambasahel@kau.edu.sa; 2 Department of Occupational Health & Air Pollution, High Institute of Public Health, Alexandria University, 165 El-Horrya Avenue, Alexandria, Egypt

**Keywords:** occupational safety and health, marine fishing, fishing vessels, young and aging fishermen, working conditions, occupational injuries, occupational illnesses, psychosocial conditions

## Abstract

Although marine fishing is one of the most hazardous occupations, research on the occupational safety and health (OSH) conditions aboard marine fishing vessels is scarce. For instance, little is known about the working conditions of vulnerable groups such as young and aging fishermen. The objective of the current paper is to study the OSH conditions of young and aging fishermen compared to middle-aged fishermen in the small- and medium-size (SM) marine fishing sector. A cross-sectional study was designed, and 686 fishermen working aboard SM fishing vessels were interviewed to collect information about their safety and health. The associations of physical and psychosocial work conditions with safety and health outcomes, e.g., injuries, illnesses and job satisfaction, are presented. The results of the current study can be utilized in the design of effective accident prevention and OSH training programs for the three age groups and in the regulation of working conditions aboard fishing vessels.

## 1. Introduction

Marine fishing has been proven to have poor occupational safety and health (OSH) records. For instance, high occupational fatality rates have been observed in many countries [1,2,3,4,5,6], and these records resulted in the classification of marine fishing as one of the most hazardous occupations [7,8,9]. In its 2014 publication on the state of world fisheries and aquaculture [10], the Food and Agriculture Organization (FAO) estimated that 39.4 million individuals were working in capture fishing in 2012, of whom 18.4 million were involved in marine fishing. The total number of marine vessels was estimated at 3.2 million, and approximately 79%, 18% and 3% of motorized marine vessels were small vessels (less than 12 m length), medium vessels (between 12 and 24 m length) and large vessels (longer than 24 m length), respectively.

Working conditions aboard small- and medium-size (SM) vessels are expected to differ from those on large vessels in various aspects. On SM vessels, most of the work is performed manually, such as throwing and hauling nets (except for bottom trawlers) and catch treatment; vessel space and accommodation are limited; work organization is immature; and many of the fishermen are informal workers. These conditions are likely to generate high physical and psychosocial demands on fishermen. Similar to physical factors, psychosocial stressors may have a negative impact on fishermen’s health and wellbeing. For instance, work-related psychosocial factors have been linked to increased risk of ill health and mortality [11].

The OSH conditions aboard commercial (or large) fishing vessels may be covered by local OSH or marshal chip regulation, whereas smaller vessels (particularly those of less than 15 m in length) are less likely to be regulated [12]. The International Labour Organization (ILO) adapted the Work in Fishing Convention in 2007 with the objective of insuring that fishers have decent work conditions on board fishing vessels. However, the Convention has not yet been put into force, as it has not yet received the required number of ratifications. The authors believe that the lack of public and regulator awareness about the OSH conditions in the remote fishing occupation is an important reason for this situation and that this lack of awareness is driven by the inadequacy and unavailability of data on occupational conditions aboard SM vessels [13].

Some interesting, yet inadequate, studies are found in the literature that endeavor to fill the gap in research on OSH aboard SM fishing vessels. However, studies of the OSH conditions of vulnerable groups in the SM fishing sector are limited, and this area is therefore not well understood. Although the global marine fishing workforce includes several vulnerable groups that may be at higher risk than others, such as younger workers, older workers, migrant workers, occasional workers and women workers, younger and older fishers are the most prevalent vulnerable groups in marine fisheries. Data are scarce on the OSH conditions of these two vulnerable occupational groups within the fishing sector.

The definition of who is considered an older person in the workforce is unclear [14]. The WHO defines aging workers as people over the age of 45, whereas the ILO defines older workers as all workers who are liable to encounter difficulties in employment and occupation because of advancement in age [15]. Other criteria have been used to define aging (sometimes older) workers, such as workers 50–64 years old [16,17]. However, the ILO defines younger workers as those who are 15–24 years of age [18].

One of the main problems in aging and work is the incompatibility between the functional capacity of a worker and the demands of the job. The work demands of a job do not usually increase with time, but work capacity typically decreases with age [15]. A decrease in physical capacity can be observed around the age of 30, followed by continued small decrements until approximately 50 years of age, when changes become more rapid [19,20,21,22,23].

Meanwhile, work injuries and illnesses among young workers are a public health concern, as young workers have rates of work injury that are typically 1.2 to two times that of older workers [24]. Younger workers have less experience and maturity in their job, which puts them at risk of overestimating their physical capacities or underestimating the safety and health risks associated with their tasks [17].

Although efforts have been made to address the working conditions of these two vulnerable groups in various occupational settings, little is known about their situation in the fishing sector, particularly aboard SM fishing vessels. The objective of the current paper is to study the OSH conditions of young and aging fishermen compared to middle-aged fishermen in the SM marine fishing sector. The study presents associations of physical and psychosocial work conditions with safety and health outcomes, e.g., injuries, illnesses and job satisfaction. The scope of the study is the SM fishing sector in Egypt, where the registered fishing workforce was reported to be approximately 65,000, and the total number is expected to be higher if informal crewmembers are included [8]. The results of the current study may also apply or be extrapolated to the same sector in other developing or underdeveloped countries.

## 2. Materials and Methods

### 2.1. Study Population and Sample Size

The current study was conducted using a cross-sectional design in El-Maaddiya seaport, located on the Egyptian Mediterranean coast. This port was selected because it receives fishing vessels from all the fishing ports along the Egyptian Mediterranean coast. The study sample of young, middle-aged and aging fishermen was randomly selected from the crew of SM fishing vessels. The fishermen were classified in the study on the basis of age: young crew were 15–24 years, middle-aged fishermen were 25–49 years, and aging crew were ≥50 years. Besides the age criterion, some other criteria were considered in the recruitment of participants. Since the working conditions vary from one vessel to another, it was necessary to involve fishermen working onboard as many vessels as possible. For this, the study team decided a maximum number of 7 fishermen (2 young, 2 aging and 3 middle-aged) from one vessel could participate. This allowed the inclusion of fishermen working on 164 fishing vessels that used various fishing gears. Furthermore, a minimum of one-year experience of the participant was used as a condition to minimize biased judgments of the inexperienced participants. The sample size of each age group with 5% precision at the 95% confidence level was calculated at 385 (i.e., total 1155). However, because of the low prevalence rate of the young and aging fishermen within the fishermen community and the limited time of the study, only 856 fishermen could be contacted. Out of them, 702 agreed to participate (82% response rate). After exclusion of the incomplete questionnaires (2.3%), the data of 686 fishermen, comprising 150 young, 122 aging and 414 middle-aged fishermen were considered. Even though there were fewer young and aging fishermen than the pre-calculated sample size, the precision was <10%.

### 2.2. Data Collection Method

The data were collected through a cross-sectional study design using interviewing techniques. Initially, focus group interviews were conducted (three groups of 10–12 fishermen) to explore the types of physical hazards; the types of accidents, injuries and illnesses; the pattern of work; and other relevant information. Based on these interviews, the study questionnaire was constructed and distributed via a pilot study with a sample of 20 fishermen comprising all age groups. Based on this pilot study, many of the questions and the possible answers were modified and the structured (formal) interview technique was decided for data collection. The field investigators (study team) spent about four months to complete the one-to-one formal interviews, which were held during fishermen free times at the sea port, cafes and other fishermen gathering places. Before deciding to participate, the interviewees were informed about the purpose of the research and that their personal data are to be confidentially treated. During the formal interviews, data were recorded on the finally approved questionnaire form by the study team. This approach was followed to minimize the potentially high exclusion rate that would result from the fishermen filling out the questionnaire, as the target group was expected to have a low educational level [8]. The questionnaire was divided into three sections. The first section included demographic information about the interviewed fishermen, such as age, education, current fishing type and years of experience. The second section collected data about the types of injuries and illnesses experienced during the past three years that resulted in days away from work, accident types, and conditions that resulted in accidents. The third section of the questionnaire was designed to collect information about working conditions, such as the physical work environment, psychosocial factors and job satisfaction.

In this study, psychosocial stress was measured according to the Demand-Control-Social Support (DCS) model. The model was initially proposed by Karasek [25] and further developed by Karasek and Theorell [26], who hypothesized that “elevation of risk with a demanding job appears only when these demands are in interaction with low control on the job”. Johnson and Hall [27] extended the model to include the social support dimension. Unlike other studies that have used the Job Content Questionnaire developed by Karasek et al. [28], the questions in the third section of the questionnaire were shortened, and binary (yes/no) answers were used to simplify the questionnaire for a targeted population with a low educational level.

### 2.3. Statistical Analysis

The logistic regression analysis is the most frequently used regression model with discrete variables [29]. Generally, logistic regression allows statistically efficient estimation of the strength and importance of the effect of each predictor variable and the interactions between them, and is able to handle multiple predictor variables [30]. Since the collected data were binary in nature (yes/no), binary logistic regression analysis was used to test the correlations among the variables of interest. For example, the associations of age group with safety and health conditions were analyzed using the Odds Ratios (ORs) which is capable of showing in which age group the association is more obvious or significant. Univariate rather than multivariate analysis was performed because the number of responses (or events) per predictor (health and safety condition) within age groups was less than 10 in about 57% of the data combinations and less than 5 in about 42% of the data combinations. If multivariate logistic analysis was used, regression coefficients would be biased in both positive and negative directions [29,31,32] (also OR would be extremely high or low).

In all the analyses, the middle-aged group was used as the reference group for comparison. The OR of a given work condition for an age group was calculated as the ratio of the odds of the association of that condition with that age group to the odds of the association of the same condition with the middle-aged group. The 95% confidence interval (CI) of the OR was calculated using the standard error of the natural logarithm of the OR as described in Morris and Gardner [33], and Bland and Altman [34]. The association was judged as significant when the value 1.0 did not exist within the 95% CI of the OR.

## 3. Results and Discussion

### 3.1. Sample Characteristics

The randomly selected sample (total 686) consisted of 21.9% young, 60.3% middle-aged and 17.8% aging fishermen. Because of the large sample size, this distribution may be considered the average age distribution within the workforce of the marine fishing sector in Egypt. Table 1 reveals that approximately 40% of the sampled young fishermen were under the age of 21 years, including 11.33% who were less than 18 years of age. The majority of these young workers reported that they had dropped out of school, and this finding is supported by the high percentage of young fishermen (78.0%) who had working experience longer than six years. Some of the remaining young workers were engaged in education, and they were working temporarily because of the low economic status of their families. Of the sampled young fishermen, 32.7% were illiterate, whereas 44.7% and 75.4% of the middle-aged and aging fishermen, respectively, were illiterate. However, 14.6%, 16.7% and 0.8% of the young, middle-aged and aging fishermen, respectively, were enrolled in high school or a university. These data reflect the poor educational qualifications of a considerable proportion of these working groups. The hazardous working conditions of the fishing sector make it an undesirable job for the educated workforce [8].

Table 1 also demonstrates that most of the interviewed fishermen were working aboard seining vessels, although this trend decreased as age group decreased. For instance, 72.7%, 62.9% and 58.2% of the young, middle-aged and aging fishermen, respectively, were working on seining gears. By contrast, the proportion of fishers working on trawling gears increased with age. The work schedule of both fishing types contributed to these trends. Seining vessels operate during the daytime, beginning in the early morning and ending in the afternoon, which was the preferred schedule for the younger workers as it gave them the opportunity to engage in other activities, such as leisure. Conversely, trawlers operate offshore for periods of 2–6 days before landing in a seaport. Although working offshore for several days in hazardous conditions is difficult for fishers of all ages, it is particularly risky for aging fishermen because they are more likely to need personal or medical care that is rarely available on these vessels.

Tobacco smoking was self-reported by 38.5%, 60.7% and 53.1% of the young, middle-aged and aging fishermen, respectively (Table 1). Similar differences among age groups are observed for the Egyptian population; however, the prevalence rates in the current study were higher than those of the Egyptian population. This could be explained by the low education level of fishermen in general. The lower prevalence rate in the younger group may be a result of developing a better awareness of the danger of smoking, e.g., through education curricula that were not available to the older age groups [35].

### 3.2. Association of Age Group with Injury Types

**Figure 1:**
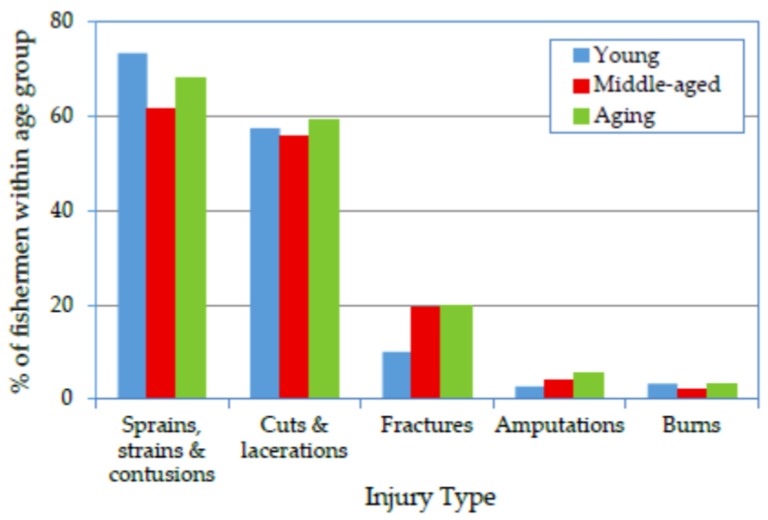
Prevalence of injury types reported by fishermen according to age group.

Figure 1 reveals that, according to the sampled fishermen, the most frequently experienced types of injuries resulting in days away from work were, in descending order, sprains, strains and contusions; cuts and lacerations; bone fractures; body part amputations; and burns. A higher proportion of young and aging fishermen than middle-aged fishermen suffered all the injury types, with the exception of bone fractures and amputations, resulting in a U-shaped relationship between age group and injury occurrence. This finding was similar to that of Jensen [36] and is in partial agreement with Lawrie et al. [37]. The logistic regression analysis revealed that the positive associations (OR > 1.0) between sprains/strains/contusions, cuts/lacerations and burns and the young and aging fishermen were higher than those observed for the middle-aged group (Table 2). However, the ORs revealed similar associations between bone fractures and both the aging and middle-aged groups, and these associations were higher than that observed for the young group. Body part amputations were more associated with the aging group than the other groups.

This U-shaped relationship could be explained by two reasons. First, the younger fishermen were less experienced, and the duration of their employment at the time of the study was not sufficient to allow them to achieve the smooth performance of a qualified worker, as illustrated by the learning curve. Second, although long experience improves safety awareness among fishermen, older workers contend with aging effects, such as the reduction of physical and mental capacity, leading to higher error and injury rates among this group than among the middle-aged group.

### 3.3. Association of Age Groups with Accident Types

**Figure 2:**
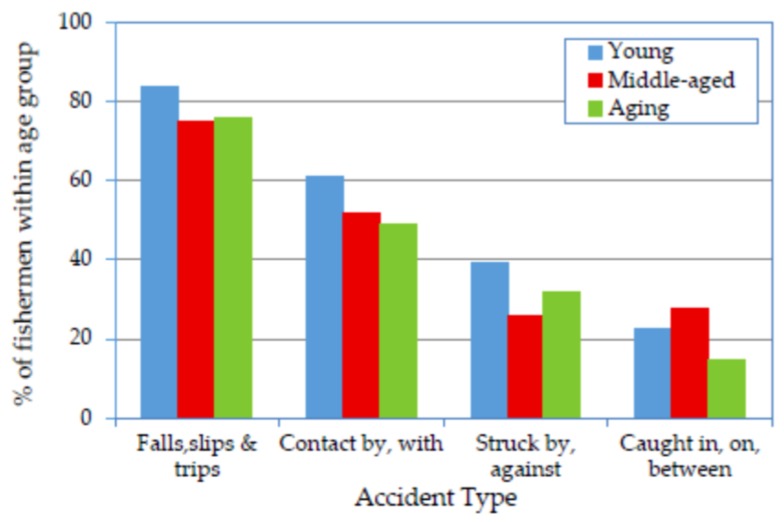
Prevalence of accident types reported by fishermen according to age group.

The types of accidents associated with the aforementioned injury types are presented in Figure 2. Undoubtedly, a high proportion of fishermen from all three age groups experienced injuries associated with the falls/slips/trips accident type, followed by the contact by/with, struck-by/against and caught-in/on/between accident types. Similar to other studies [9,12,38,39], high occurrence rate of falls/slips/trips accidents was observed. Both falls/slips/trips and struck-by/against accidents formed a U-shaped relationship with age group. The logistic regression analysis revealed that the associations between all the accident types and the young group were higher than those observed for the aging group, and ORs for the young relative to the aging fishermen were >1.0 (Table 2). The same pattern was observed when comparing the association between accident type and both the young and the middle-aged groups, except for the caught-in/on/between accident type, whose association with the middle-aged was slightly higher. The ORs also indicated that the associations between the falls/slips/trips and the contact by/with accident types and the aging group were comparable to those of the middle-aged group. However, the association with the struck-by/against accident type was higher for the aging group than the middle-aged group, and the association with the caught-in/on/between accident type was lower for the aging group than for the middle-aged group.

Many reasons may have increased the occurrence rate of the falls/slips/trips accident type. The inexperience of the young fishermen and their more frequent movements aboard may have rendered them vulnerable to this accident type. Additionally, seasickness is known to be more prevalent among young crewmembers than others, which may have increased the young crewmembers’ sense of instability and consequently increased the possibility of suffering a fall accident. By contrast, the significant changes in balance and body control resulting from aging [40] may have caused more body sway [41] among the aging group, rendering them highly vulnerable to fall accidents. However, the extensive experience of the aging group may have aided them in developing protective measures, such as shortening walking periods or standing during rest and idle periods. This may be the reason for the slight difference between the aging and middle-aged groups in terms of the occurrence of falls/slips/trips accidents.

The struck-by/against accident type exhibited a U-shaped relationship with age group, perhaps because the young fishermen paid less attention to moving or fixed objects due to their inexperience and because of deteriorated vision [40] and slow reactions [42] among the aging fishermen. However, the caught-in/on/between accident type exhibited an inverse U-shaped relationship, mainly because this type of accident is associated with the most dangerous tasks aboard SM fishing vessels, and these tasks were assigned to the middle-aged fishermen who were well-experienced and physically fit to perform them.

### 3.4. Association of Age Groups and Accident Conditions with Accident Types, Injury Types

**Figure 3:**
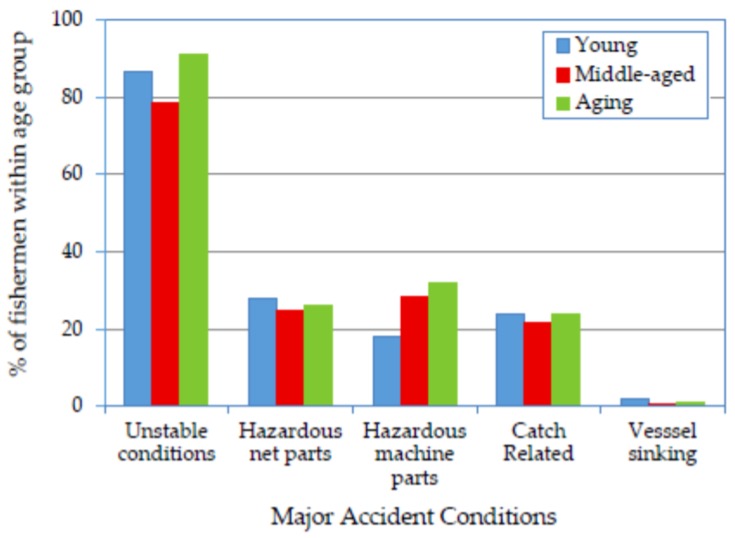
Prevalence of major accident conditions reported by fishermen according to age group.

Figure 3 reveals that unstable conditions (e.g., rocking vessel motion and wet deck) was the accident condition most frequently reported by the fishermen, followed by hazardous net parts, hazardous machine parts, catch-related and, finally, vessel sinking. Unstable conditions, hazardous net parts and catch-related factors exhibited U-shaped relationships with age group, whereas the association of hazardous machine parts increased with age group. The ORs in Table 2 support these findings. The middle-aged group had both enough experience and physical fitness to be the group least affected by the unstable conditions on SM vessels. Furthermore, these two characteristics assisted the middle-aged fishermen in developing better reaction times to accidents associated with hazardous net parts, machine parts and catch. The lower association of the young group with hazardous machine parts was attributed primarily to the fact that most of the tasks related to machinery (engine, winch and tiller) are assigned to experienced fishermen because a vessel’s success is highly dependent on these tasks. Figure 3 also demonstrates that some of the accidents resulted from vessel sinking. However, the number of cases was too small to permit an interpretation of these data.

Unstable conditions were common causes of falls [8]. The logistic regression of the association of unstable conditions with falls/slips/trips accidents (Table 3) confirmed a high association for all age groups collectively (OR = 21.2). The association of unstable conditions with falls/slips/trips accidents was more evident in the young group. Surprisingly, the same association was least evident in the aging group despite the higher significance found for the association of unstable conditions with the aging group relative to the other two age groups (Table 2). These findings suggest that some of the aging fishermen mentioned unstable conditions as an accident-related factor even when they had not actually experienced a related accident. While the association between unstable conditions and falls/slips/trips accidents is understood, the significance of the association between unstable conditions and struck-by/against accidents could be attributed to storing the majority of a vessel’s fishing gear on the deck, including fixed and loose parts. Unstable vessel conditions may have increased the possibility that the fishermen would be struck by or against these parts.

The association of unstable conditions with injury type was significant in the following order: sprains/strains/contusions (OR = 49.4), cuts/lacerations (OR = 20.4) and bone fractures (OR = 16.9). These injury types were commonly associated with the falls/slips/trips and struck-by/against accident types. The regression analysis revealed that most of the reported sprains/strains/contusions and bone fractures occurred because of unstable conditions.

Table 3 reveals strong associations between the contact-by/with accident type and hazardous net parts (OR = 10.0), hazardous machine parts (OR = 10.6) and catch-related (OR = 30.5), regardless of age group. The strong association of these accident conditions with the contact accident type could be explained by the high dependence on manual material handling for work aboard SM fishing vessels. The process of throwing and hauling (pulling) nets involves contact with parts that can easily cause wounds or skin cuts (OR = 32.4), such as sheaves, rings and wires. Handling machinery parts caused some contact injuries such as burns (OR = 4.4) and cuts (OR = 8.6). Manual treatment of the catch and fish boxes (mostly wooden) was the most important cause of contact accidents (OR = 36.5). The interviewed fishermen mentioned that injuries caused by contact with some types of fish, jelly fish and crustaceans were very common and resulted in days away from work. Consistent with this, strong associations were found between cuts/lacerations and hazardous net parts (OR = 32.4), hazardous machine parts (OR = 8.6) and catch-related (OR = 36.5). The associations of all three accident conditions with the contact accident type were higher for the young group than for the other groups. The short experience of the younger crewmembers rendered them more vulnerable to this type of accident. By contrast, it is likely that the aging fishermen protected themselves by avoiding tasks related to the contact-by/with type of accident whenever possible.

The associations of both hazardous net parts and hazardous machine parts with both the struck-by/against and the caught-in/on/between accident types were significant, causing portions of the sprains/strains/contusions and cuts/lacerations injuries. Furthermore, the struck-by/against and caught-in/on/between accident types associated with hazardous machine parts were major contributors to bone fractures (OR = 3.2) and amputations (OR = 8.5). The critical machinery parts associated with these injury types were winch, motor conveyor and gears, propeller, motor hand crank, and tiller arm. Handling these machinery parts requires long experience, leading a stronger association of the aging and middle-aged groups than the young group with the resulting injury types (bone fractures and amputations).

### 3.5. Association of Age Groups and Work Conditions with Illnesses

**Figure 4:**
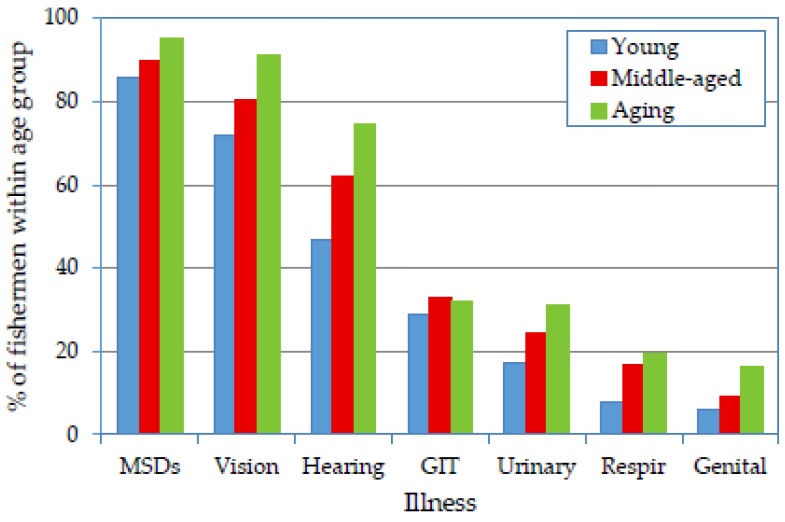
Prevalence of major illnesses reported by fishermen according to age group.

Although the interviewed fishermen mentioned many illnesses, only the most prevalent types were analyzed. Figure 4 reveals that musculoskeletal disorders (MSDs) and vision, hearing, gastrointestinal (GIT), urinary tract, respiratory tract and genital tract disorders (mainly prostatitis) were the most prevalent illnesses in descending order. Apart from GIT disorders, the aging group exhibited the highest rates of illness occurrence, followed by the middle-aged and young fishermen. This trend might be expected based on numerous studies [8], and important causes of the trend may be the risk factors associated with aging workers, such as longer exposure to occupational hazards and greater risk of developing long-term or chronic illnesses or disabilities [17].

**Figure 5:**
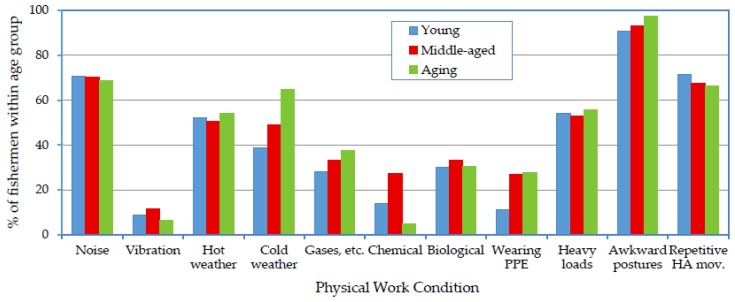
Exposure of fishermen to physical work conditions according to age group.

The association of age group with the most prevalent self-reported illnesses is displayed in Table 2. The health condition of the fishermen was expected to be affected by many environmental work conditions, which are presented in Figure 5. In addition, the logistic regression for the associations of environmental conditions and age groups with self-reported illnesses is presented in Table 4.

Table 2 reveals that there were variations in the significance of the associations between age group and illness. Relative to the middle-aged group, the associations of the young group were significant only for MSDs and vision, hearing and respiratory problems. Conversely, the associations of the aging group (relative to the middle-aged) were significant only for vision, hearing and genital disorders. This is an indication that the working conditions aboard SM fishing vessels were so hazardous that all age groups were vulnerable to developing occupational health problems.

Many conditions existing on SM fishing vessels contributed to the high prevalence of MSDs among all three age groups [8]. For instance, the majority of the fishermen in each age group mentioned that they were exposed to hazardous physical environmental conditions such as heavy load handling, awkward postures and cold weather (Figure 5). The ORs revealed a significant association of these conditions with MSDs, which was more evident in the aging group (Table 4).

The occurrence rate of MSDs (Figure 4) among the young group (86.0%) was extremely high, although it was lower than that observed for the middle-aged and aging fishermen. Young workers may be at risk of developing MSDs because they are more likely to be given the most challenging physical tasks because of their young age and relatively good physical health [17]. Furthermore, as mentioned earlier, the majority of the young fishermen worked aboard seining vessels (Table 1). The work on this type of fishing gear was found to depend on manual pulling (hauling) of nets in a bent-back posture for long periods, resulting in a high risk of developing MSDs.

High occurrence rates were observed for vision problems. Many factors may contribute to vision problems among marine fishermen, such as sunlight, staring at the sea’s surface, salt water and wind [8]. The combined effect of many concurrent factors might have resulted in the high occurrence rate of vision problems among all age groups. Table 4 shows a significant association of environmental conditions, such as vibration, hot conditions and biological hazards, with vision problems. Working offshore in hot weather conditions involves exposure to direct sunlight, which is a source of UV radiation. Additionally, biological hazards include contact of the eye with flying bioparticles resulting from net cleaning by shaking and from splashing during jelly fish handling.

Hearing problems were expected for all the age groups due to continuous exposure to engine noise on SM fishing vessels [13,43]. The occurrence rate was highest among the older fishermen because of the effect of aging, e.g., in the form of presbycusis. Figure 5 indicates a high perception of noise problems among the three age groups: 70.7%, 70.3% and 68.9% of the young, middle-aged and aging fishermen, respectively, mentioned that they were exposed to high noise levels. The associations of both noise exposure and age group with hearing problems were significant (Table 4).

The occurrence rates of GIT disorders were very similar among the three age groups (Figure 4), and their associations with age group were therefore insignificant (Table 2). This is an indication that the young and middle-aged fishermen were subjected to stressors that increased their occurrence rates to a level similar to that of the aging group. Table 4 indicates that the associations of working conditions such as noise, vibration, hot conditions, cold conditions, biological hazards and smoking with GIT disorders were generally significant. However, there were no significant differences among the age groups. Seasickness (caused by unstable conditions) may be an additional factor since it is typically associated with nausea or vomiting.

Other major illnesses such as urinary, respiratory and genital tract disorders were prevalent in ascending order from the younger to the older age groups (Figure 4). However, the occurrence of these illnesses may be higher in the fisher population than in the average population because of the poor environmental conditions in which fishermen work. For instance, significant associations were found between working in cold conditions and these three illnesses, and the differences among the age groups were generally insignificant (Table 4).

Factors such as working in hot conditions, working in cold conditions and heavy load handling were significantly associated with urinary tract problems. High sweating rate with low water intake in hot conditions could result in urinary tract problems. Heavy load handling was also found to adversely affect the lower urinary tract. Furthermore, the majority of the interviewed fishermen reported that they did not wear personal protective equipment (Figure 5), particularly during the cold winter season and, therefore, remained wet for long periods, a condition that likely increased the occurrence rate of urinary tract disorders. Table 4 indicates that working in cold conditions and heavy load handling were also significantly associated with genital tract disorders. However, a potential relationship between urinary and genital disorders should not be excluded.

Respiratory problems were significantly associated with working in cold conditions, gases and smoke, and biological hazards (Table 4). Figure 5 reveals that 28.0%, 33.3% and 37.7% of the young, middle-aged and aging groups, respectively, reported that they were exposed to gases, fumes and smoke. Most of the young fishermen attributed their exposure to smoking by others, whereas most of the middle-aged and aging fishermen attributed their exposure to engine exhaust gases. Not using protective suits during cold weather may have increased the respiratory problems occurrence.

Table 4 indicates that smoking was significantly associated with GIT, urinary, respiratory and genital problems and that the association was more significant with respiratory problems. The associations of smoking with the remaining health problems were insignificant.

### 3.6. Association of Age Groups and Psychosocial Work Conditions with Job Satisfaction

**Figure 6:**
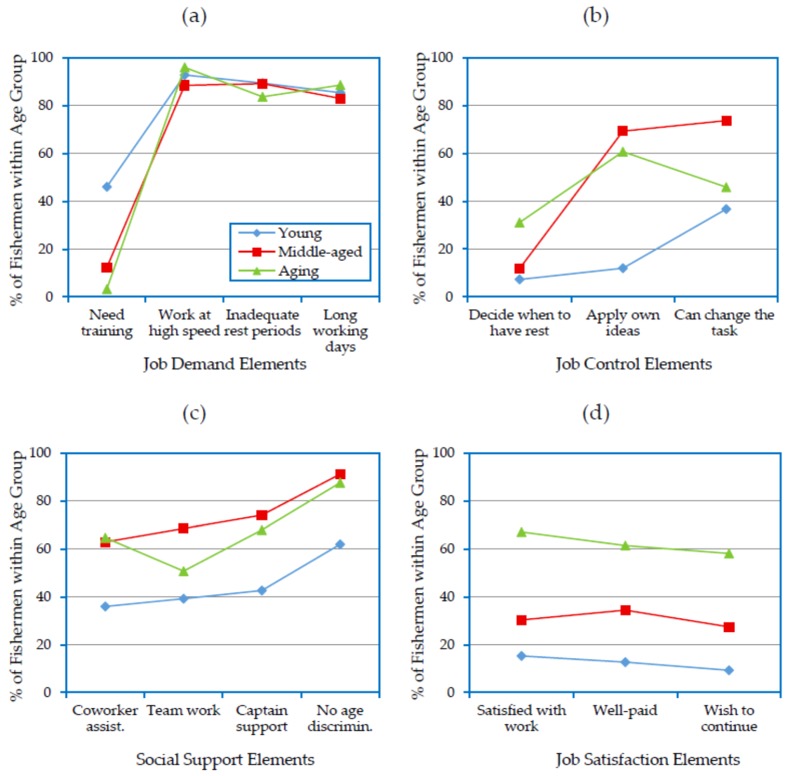
Factors affecting job content among fishermen age groups: (**a**) job demand; (**b**) job control; (**c**) social support; and (**d**) job satisfaction.

Figure 6 shows the psychosocial work conditions aboard SM fishing vessels for all age groups. Figure 6a indicates that the majority of fishermen believed that their jobs were psychologically demanding, regardless of age. Except for the need for training, there were slight differences among the age groups in terms of their perception of other job demand factors, i.e., working at high speed, inadequate rest periods and long working days. This is mainly because most of the tasks aboard SM vessels are manual in nature, and crewmembers spend long periods to complete a hunting cycle. A high targeted number of cycles per day resulted in limited time for rest, high speed work and long working days. Despite the similar perception of job demand factors among the age groups, the potential effect of the job demand factors on job satisfaction varied among them. Table 5 reveals an overall significant negative association of job demand factors with job satisfaction (OR = 0.04–0.2). The ORs indicated that the negative association was more evident among the young fishermen (e.g., OR = 0.3–0.6), whereas it was least observable among the aging group (e.g., OR = 4.3–7.4).

Figure 6b suggests that the middle-aged and aging groups had higher levels of job control than the young group. The short experience of the young group compared to the other groups provided less opportunity of making decisions. The exception to this was the young fishermen who were vessel owners or partners. They had better opportunity to make decisions and consequently to control their jobs. The middle-aged group members were also found to have the highest rates of applying their own ideas and changing their tasks. The work aboard fishing vessels was found to depend mainly on the middle-aged group members (they were about 60% of the study sample). The middle-aged crewmembers had enough experience and physical fitness to handle the most important tasks, and this encouraged vessel owners to grant them more authority and power than the other age groups. The aging group also had some control, although less than the middle-aged group, except for rest periods where they had more control than the other groups. It appeared that many of fishermen and vessels owners understood that the health status of aging fishermen required them to take more rest periods than the others. However, the aging group’s control over rest periods was found to be much less than needed given the high physical demand of the job. Table 5 reveals an overall positive association of job control factors with job satisfaction. The association was stronger for the factor “can decide when to rest” (OR = 15.9) than for the other two factors (OR = 2.0 and 2.3). This is in accordance with the previously mentioned significant negative association of job demand factors with job satisfaction. In all cases, the association of job control factors with job satisfaction was more observable in the aging group and less observable in the young group.

The three age group members’ perception of social support factors is presented in Figure 6c, which reveals that the middle-aged group had the best social conditions and that the young group had the least social support. Many reasons may have caused these differences in social conditions among the three age groups aboard SM fishing vessels. Since the middle-aged group constitutes the major and most vital work group in the fishermen community, its members are more likely to receive support from vessel owners and captains. This is reflected in the middle-aged fishermen’s positive impression that they were not subject to age discrimination. Furthermore, the higher level of job control reported by the middle-aged group may have increased their ability to develop teamwork and obtain assistance from coworkers. Conversely, the values and attitudes developed within the fishermen community towards the aging group may have had a role in elevating this group’s perception of social support factors. Unlike the other age groups, the young fishermen reported low levels of social support. The low educational level among the fishermen (Table 1) may have prevented vessel owners, captains and older crew from developing the management skills necessary to deal with young fishermen and facilitate their engagement in the vessel team. Additionally, most of the older fishermen had a traditional belief that younger fishermen are less likely to develop the skills necessary to continue working unless they suffer hard times on the job. The logistic regression found a very similar positive association of all the social support factors with job satisfaction in general (Table 5). The significance of the association was highest for the aging group and lowest for the young group.

The work satisfaction elements are presented in Figure 6d. Interestingly, the aging group reported the highest rates for all the job satisfaction elements, followed by the middle-aged group and the young group. The variations among the three age groups were significant, and the ORs were 3.0–4.7 and 0.3–0.4 for the aging and young groups, respectively, relative to the middle-aged group (Table 2). Despite having better job control and social support than the other age groups, the middle-aged group did not report the highest level of job satisfaction. This indicates that job demand had a greater impact on job satisfaction than job control and social support among the middle-aged group. This is supported by the ORs from the logistic regression analysis of the association of job demand elements with job satisfaction (Table 5), which indicated that the negative impact of job demand on job satisfaction was more significant in the middle-aged group than the aging group.

Unlike the middle-aged group, job control and social support had a more favorable impact on job satisfaction among the aging group members. Furthermore, the older fishermen viewed this occupation differently from the young and middle-aged fishermen. Most of the aging fishermen (75.4%) were illiterate, and most of them (71.3%) had spent over 40 years in this job (Table 1) without opportunities to obtain other jobs with better working conditions; therefore, they may have been unable to compare their working conditions to those of other jobs. Consequently, the older fishermen were more satisfied with their working conditions than the young and middle-aged groups who may have had or at least had access to information about jobs with better working conditions.

The most notable finding in accordance with the DCS model was the level of job satisfaction among the members of the young group, who they reported high job demand, low decision latitude (job control) and low social support. According to the DCS model, the young group members were exposed to psychosocial conditions that resulted in high strain and isolation [27] and had the greatest negative effect on their wellbeing [44]. Consequently, their job satisfaction was negatively affected [25].

### 3.7. Association of Age Group and Psychosocial Work Conditions with Musculoskeletal Disorders

In the previous sections, the associations of physical work conditions with accident/injury types and illnesses were analyzed. Many studies also found associations of psychosocial factors with safety and health problems. For instance, MSDs have been linked to psychosocial work environment in several studies [45,46,47,48,49,50]. Table 5 presents the association of psychosocial work conditions aboard SM fishing vessels with MSDs, revealing positive associations of the job demand elements with MSDs. The association was slightly less apparent among the aging group than among the middle-aged group (OR = 0.8), whereas it was significantly less evident among the young group (OR = 0.2–0.3). However, most of the social support elements (except for age discrimination) and one job control element (can decide when to have rest) had negative associations with the occurrence of MSDs, with ORs less than 1.0 (Table 5). In contrast to the job demand elements, the favorable impact of social support and decision elements on the occurrence of MSDs was more evident among the young group (OR = 0.2–0.3) than the aging group (OR = 0.8–1.0), both relative to the middle-aged group. 

Overall, there was negative association of work satisfaction with the occurrence of MSDs within the study sample of fishermen (Table 5). This negative association was more observable among the young group (OR = 0.2), whereas it was least evident among the aging group (OR = 1.1), both relative to the middle-aged group.

These results suggest that the high prevalence of MSDs within the young and middle-aged groups (Figure 4) was not related solely to the hard physical work conditions (shown in Figure 5). The psychosocial work conditions may have had a role in elevating the prevalence of MSDs among the most physically fit age groups. However, more weight could be given to the physical work conditions and aging effects as contributors to MSDs within the aging group at the expense of psychosocial conditions.

### 3.8. Limitations of the Study

There are some limitations that should be considered in the current study. The study was of the cross-sectional type, which did not allow for solid statements on the causative relationship between an environmental or psychosocial condition and an illness or job satisfaction [51]. This did not apply for the relationships among injury type, and accident type and condition since the relationship in this case was not of the long-term type. To minimize the impact of this limitation on the reliability of the study results, intensive group discussions were conducted with the fishermen to collect as much information as possible about the occupation, work patterns, possible causes of illnesses and accidents, fishermen lifestyle, etc., so that the results could be realistically interpreted. Another possible problem with the cross-sectional study was the “healthy worker” effect, which means that the workers with ill health might have exited the workforce [52] and, hence, there OSH data would be unavailable. For these, it is recommended that a longitudinal study be conducted to get more accurate information about the causes of reported health problems and the psychosocial health outcomes. 

Another limitation was that the low educational level of the majority of the interviewed fishermen had partial impact on the questionnaire design, particularly the number of questions and the answer format. Based on a preliminary sample, some questions of the job content section were omitted and the answers to most of the questions were changed to the binary format. This affected the selection of the regression analysis method. Thus, binary logistic regression was used, and the significance and strength of associations were analyzed. A 5-point Likert scale would result in using another regression model and more deep analysis. Furthermore, the use of univariate logistic regression could result in regression coefficient bias due to the effect of variables (predictors) interactions and confounding. Again, the group discussions gave valuable information that supported the significant associations resulting from the binary logistic regression analysis. However, further studies with larger sample size within each age group will allow for accurate multiple regression analysis.

Because of the absence of OSH records (i.e., injuries, health, and compensation) the study results were based on self-reported injuries, illnesses and psychosocial conditions. This adds to the uncertainty of the results because of possible bias.

## 4. Conclusions

Studies of the working conditions of vulnerable age groups, e.g., young and aging workers, in the fishing sector are limited and are not easily found in the literature. The current study presents a relatively detailed investigation of the OSH conditions of these two vulnerable age groups compared to the middle-aged group in the SM marine fishing sector. Although the results of the current study demonstrate, as in other sectors, a U-shaped relationship between age and injury occurrence and a trend of increasing illness with age, this study is unique in presenting a detailed analysis of the reasons for the occurrence of injuries and illnesses among the three age groups in the SM fishing industry, particularly accident types and unsafe conditions. Furthermore, psychosocial working conditions were discussed in light of the DCS model. Based on overall work satisfaction, the young age group was found to be the most vulnerable to job stress, whereas the aging group was the least vulnerable. Although the middle-aged group had better job control and better social support, they were not the most satisfied group. The associations of the psychosocial work conditions with MSD occurrence suggest that the former may be a contributor (in addition to physical conditions) to the occurrence of MSDs, particularly among young fishermen.

The presented associations of work conditions and health and safety outcomes aboard SM fishing vessels could be utilized in the design of effective accident prevention and OSH training programs for the three age groups and in the regulation of working conditions aboard fishing vessels. Therefore, further studies of interventions to enhance both physical and psychosocial work conditions are recommended.

## Figures and Tables

**Table 1:** Demographic characteristics of the young, middle-aged and aging fishermen sample.

Characteristic	% Fishermen			Characteristic	% Fishermen		
	Young (15–24 Years) *n* = 150	Middle-Aged (25–49 Years) *n* = 414	Aging (≥50 Years) *n* = 122		Young (15–24 Years) *n* = 150	Middle-Aged (25–49 Years) *n* = 414	Aging (≥50 Years) *n* = 122
***Age group***				***Years on job***			
<18 years	11.33			<3 years	2.0		
18–21 years	29.33			3–6 years	20.0		
21–25 years	59.33			≥6 years	78.0		
25–30 years		29.5		<10 years		3.4	
30–40 years		40.1		10–20 years		37.7	
40–50 years		30.4		≥20 years		58.9	
50–55 years			38.5	<20 years			2.5
55–60 years			32.0	20–40 years			26.2
≥60 years			29.5	≥40 years			71.3
***Fishing gear***				***Education level***			
Seiners	72.7	62.9	58.2	Illiterate	32.7	44.7	75.4
Trawlers	14	17.1	20.5	Read and write	52.7	38.6	23.8
All others	13.3	20.0	21.3	High school	13.3	15.7	0.8
***Smoking***				University	1.3	1.0	0
Tobacco smokers	38.5	60.7	53.1				

**Table 2:** Logistic regression analysis of the association of age groups (as predictor) with accident conditions, accident types, injury types and self-reported illnesses (as responses).

Safety and Health Condition	Odds Ratios (95% CI) Relative to the Middle-Aged	
	Aging	Young
***Injury types***		
Sprains, strains and contusions	1.3 (0.9, 2.0)	1.7 (1.1, 2.6)
Cuts and lacerations	1.2 (0.8, 1.7)	1.1 (0.7, 1.6)
Bone fractures	1.0 (0.6, 1.7)	0.5 (0.3, 0.8)
Amputations	1.5 (0.6, 3.8)	0.7 (0.2, 2.1)
Burns	1.7 (0.5, 5.8)	1.8 (0.6, 5.4)
***Accident types***		
Falls, slips and trips	1.1 (0.7, 1.7)	1.8 (1.1, 2.9)
Contact (by or with)	0.9 (0.6, 1.4)	1.5 (1.0, 2.2)
Struck (by or against)	1.3 (0.9, 2.1)	1.9 (1.3, 2.8)
Caught (in, on or between)	0.5 (0.3, 0.8)	0.8 (0.5, 1.2)
***Accident conditions***		
Unstable vessel	2.8 (1.4, 5.4)	1.8 (1.1, 3.0)
Hazardous net parts	1.1 (0.7, 1.7)	1.2 (0.8, 1.8)
Hazardous machine parts	1.2 (0.8, 1.9)	0.6 (0.4, 0.9)
Catch-related (fish and boxes)	1.1 (0.7, 1.8)	1.2 (0.7, 1.8)
Vessel sinking	1.7 (0.2, 18.9)	4.2 (0.7, 25.4)
***Illnesses***		
MSDs	0.8 (0.3, 2.1)	0.3 (0.1, 0.5)
Vision problems	2.4 (1.2, 4.7)	0.6 (0.4, 0.9)
Hearing problems	1.8 (1.1, 2.8)	0.5 (0.4, 0.8)
Gastro-intestinal problems	1.0 (0.6, 1.5)	0.8 (0.5, 1.2)
Urinary tract problems	1.4 (0.9, 2.2)	0.6 (0.4, 1.0)
Respiratory problems	1.2 (0.7, 2.1)	0.4 (0.2, 0.8)
Genital problems (mainly prostatitis)	2.0 (1.1, 3.6)	0.7 (0.3, 1.4)
***Work satisfaction***		
Satisfied with work	4.7 (3.0, 7.2)	0.4 (0.3, 0.7)
Well-paid for work	3.0 (2.0, 4.6)	0.3 (0.2, 0.5)
Wish to continue in work	3.7 (2.4, 5.6)	0.3 (0.2, 0.5)

**Table 3:** Logistic regression analysis of the association of both age group and major accident conditions (as predictors) with accident types and injury types (as responses) (only important associations are displayed for table simplicity and readability).

**Major Accident Condition**	**Age Group**	**Odds Ratios (95% CI) for the Association with Accident Type**				
		**Falls, Slips and Trips**	**Contact** **(by or with)**	**Struck** **(by or against)**	**Caught** **(in, on or between)**	
Unstable vessel	Overall ^**1**^ Aging ^**2**^ Young ^**2**^	21.2 (10.0, 34.7) 0.6 (0.4, 1.1) 1.5 (0.8, 2.6)		3.4 (1.9, 6.0) 1.2 (0.8, 1.9) 1.7 (1.2, 2.6)		
Hazardous net parts	Overall Aging Young		10.0 (6.2, 16.2) 0.9 (0.6, 1.3) 1.5 (1.0, 2.3)	2.6 (1.8, 3.7) 1.3 (0.6, 2.1) 1.9 (1.2, 2.8)	10.6 (7.1, 16.1) 0.4 (0.2, 0.6) 0.6 (0.4, 1.1)	
Hazardous machine parts	Overall Aging Young		10.6 (6.6, 16.9) 0.8 (0.5, 1.3) 2.0 (1.3, 2.9)	2.5 (1.7, 3.5) 1.3 (0.8, 2.1) 2.1 (1.4, 3.2)	7.2 (4.8, 10.6) 0.4 (0.2, 0.7) 1.0 (0.6, 1.6)	
Catch-related (fish and boxes)	Overall Aging Young		30.5 (14.0, 66.6) 0.8 (0.5, 1.3) 1.5 (1.0, 2.3)			
**Major Accident Condition**	**Age Group**	**Odds Ratios (95% CI) for the Association with Injury Type**				
		**Sprains, Strains and Contusions**	**Cuts and Lacerations**	**Bone Fractures**	**Amputations**	**Burns**
Unstable vessel	Overall Aging Young	49.4 (23.4, 104.4) 0.9 (0.5, 1.4) 1.5 (0.9, 2.5)	20.4 (10.7, 39.0) 0.8 (0.5, 1.3) 0.9 (0.6, 1.3)	16.9 (4.1, 69.4) 0.8 (0.5, 1.4) 0.4 (0.2, 0.7)		
Hazardous net parts	Overall Aging Young	10.0 (5.5, 18.1) 1.3 (0.9, 2.1) 1.7 (1.1, 2.7)	32.4 (14.9, 70.5) 1.2 (0.7, 1.8) 1.0 (0.6, 1.5)			
Hazardous machine parts	Overall Aging Young	2.1 (1.4, 3.1) 1.3 (0.8, 2.0) 1.9 (1.2, 2.8)	8.6 (5.4, 13.8) 1.0 (0.7, 1.7) 1.3 (0.9, 2.0)	3.2 (2.1, 4.8) 1.0 (0.6, 1.6) 0.5 (0.3, 0.9)	8.5 (3.5, 20.5) 1.4 (0.6, 3.6) 0.9 (0.3, 2.8)	4.4 (1.6, 11.8) 1.6 (0.5, 5.6) 2.1 (0.7, 6.8)
Catch-related (fish and boxes)	Overall Aging Young		36.5 (14.7, 90.4) 1.1 (0.7, 1.8) 1.0 (0.7, 1.6)			

**^1^** Association of major accident condition with accident or injury type for all age groups collectively; **^2^** Relative to the middle-aged group.

**Table 4:** Logistic regression analysis of the association of both age group and physical environmental conditions (as predictors) with major self-reported illnesses (as response) (only important associations are displayed for table simplicity and readability).

**Major Illness**	**Age Group**	**Odds Ratios (95% CI)**				
		**Noise**	**Vibration**	**Hot Conditions**	**Cold Conditions**	**Gases and Smoke**
MSDs	Overall ^**1**^ Aging ^**2**^ Young ^**2**^		4.8 (0.7, 36.0) 0.9 (0.3, 2.2) 0.3 (0.1, 0.5)		2.9 (1.4, 5.9) 0.7 (0.3, 1.8) 0.3 (0.1, 0.6)	
Vision problems	Overall Aging Young		4.4 (1.6, 12.4) 2.5 (1.3, 5.0) 0.6 (0.4, 1.0)	2.6 (1.7, 3.9) 2.4 (1.2, 4.7) 0.6 (0.4, 0.9)		
Hearing problems	Overall Aging Young	2.5 (1.8, 3.6) 1.9 (1.2, 3.0) 0.5 (0.4, 0.8)				
Gastrointestinal disorders	Overall Aging Young	5.5 (3.4, 8.7) 1.0 (0.6, 1.5) 0.8 (0.5, 1.2)	1.9 (1.1, 3.2) 1.0 (0.6, 1.5) 0.8 (0.5, 1.2)	2.6 (1.9, 3.6) 0.9 (0.6, 1.4) 0.8 (0.5, 1.2)	3.5 (2.5, 5.0) 0.8 (0.5, 1.2) 0.9 (0.6, 1.4)	
Urinary problems	Overall Aging Young			1.9 (1.3, 2.8) 1.4 (0.9, 2.2) 0.6 (0.4, 1.0)	12.6 (7.6, 21.1) 1.0 (0.6, 1.7) 0.8 (0.5, 1.3)	
Respiratory tract problems	Overall Aging Young				6.0 (3.5, 10.3) 1.0 (0.6, 1.7) 0.5 (0.3, 1.0)	1.8 (1.2, 2.8) 1.2 (0.7, 2.0) 0.4 (0.2, 0.9)
Genital tract problems	Overall Aging Young				24.4 (7.6, 78.7) 1.6 (0.8, 2.9) 0.8 (0.4, 1.8)	
**Major illness**	**Age Group**	**Odds Ratios (95% CI)**				
		**Biological Hazards**	**Heavy Loads**	**Awkward Postures**	**Repetitive Hand/Arm Motion**	**Smoking**
MSDs	Overall Aging Young		4.5 (2.2, 9.4) 0.8 (0.3, 2.1) 0.2 (0.1, 0.5)	3.4 (1.4, 8.0) 0.8 (0.3, 2.0) 0.3 (0.1, 0.5)	0.6 (0.3, 1.3) 0.8 (0.3, 2.1) 0.3 (0.1, 0.5)	
Vision problems	Overall Aging Young	3.7 (2.2, 6.3) 2.6 (1.3, 5.0) 0.6 (0.4, 1.0)				
Hearing problems	Overall Aging Young					1.3 (0.9, 1.7) 1.8 (1.2, 2.9) 0.6 (0.4, 0.8)
Gastrointestinal disorders	Overall Aging Young	2.7 (1.9, 3.8) 1.0 (0.6, 1.5) 0.8 (0.5, 1.3)				2.1 (1.5, 2.9) 1.0 (0.6, 1.6) 1.0 (0.6, 1.4)
Urinary problems	Overall Aging Young		2.3 (1.6, 3.3) 1.4 (0.9, 2.2) 0.6 (0.4, 1.0)			1.6 (1.1, 2.4) 1.5 (0.9, 2.3) 0.7 (0.4, 1.2)
Respiratory tract problems	Overall Aging Young	1.8 (1.1, 2.7) 1.2 (0.7, 2.1) 0.4 (0.2, 0.8)				9.9 (5.0, 19.5) 1.4 (0.8, 2.5) 0.6 (0.3, 1.2)
Genital tract problems	Overall Aging Young		3.0 (1.6, 5.3) 2.0 (1.1, 3.6) 0.6 (0.3, 1.4)			1.7 (1.0, 3.0) 2.1 (1.2, 3.8) 0.7 (0.3, 1.6)

**^1^** Association of physical environmental conditions with major illnesses for all age groups collectively; **^2^** Relative to the middle-aged group.

**Table 5:** Logistic regression analysis of the association of age groups and psychosocial conditions (as predictors) with work satisfaction and MSDs (as responses).

Psychosocial Conditions	Age Group	Odds Ratios (95% CI)	
		Work Satisfaction	MSDs
***Job demand***			
Feel need training	Overall ^**1**^ Aging ^**2**^ Young ^**2**^	0.2 (0.1, 0.3) 4.3 (2.8, 6.7) 0.6 (0.4, 1.1)	1.4 (0.6, 3.1) 0.8 (0.3, 2.2) 0.2 (0.1, 0.5)
Working at high speed	Overall Aging Young	0.02 (0.01, 0.05) 6.6 (4.2, 10.4) 0.4 (0.2, 0.7)	2.2 (0.9, 5.4) 0.8 (0.3, 2.0) 0.3 (0.1, 0.5)
Have inadequate rest periods	Overall Aging Young	0.02 (0.01, 0.05) 5.3 (3.3, 8.4) 0.3 (0.1, 0.5)	1.2 (0.5, 3.0) 0.8 (0.3, 2.2) 0.3 (0.1, 0.5)
Have long working days	Overall Aging Young	0.04 (0.02, 0.06) 7.4 (4.7, 11.9) 0.3 (0.2, 0.6)	2.0 (1.0, 4.2) 0.8 (0.3, 2.1) 0.3 (0.1, 0.5)
***Job control***			
Can decide when to rest	Overall Aging Young	15.9 (8.6, 29.4) 3.9 (2.4, 6.2) 0.4 (0.2, 0.7)	0.5 (0.2, 1.1) 1.0 (0.4, 2.6) 0.3 (0.1, 0.5)
Can apply your own ideas	Overall Aging Young	2.3 (1.6, 3.5) 5.4 (3.4, 8.4) 0.7 (0.4, 1.2)	1.3 (0.6, 2.8) 0.8 (0.3, 2.2) 0.3 (0.1, 0.7)
Can change tasks when you wish	Overall Aging Young	2.0 (1.4, 3.0) 6.1 (3.8, 9.7) 0.5 (0.3, 0.9)	1.1 (0.6, 2.1) 0.9 (0.3, 2.3) 0.3 (0.1, 0.6)
***Social support***			
Easy to get assistance from coworkers	Overall Aging Young	1.9 (1.4, 2.8) 4.8 (3.1, 7.5) 0.5 (0.3, 0.8)	0.7 (0.3, 1.3) 0.8 (0.3, 2.2) 0.2 (0.1, 0.5)
Working as a team	Overall Aging Young	1.6 (1.1, 2.3) 5.2 (3.3, 8.1) 0.5 (0.3, 0.8)	0.7 (0.4, 1.4) 0.8 (0.3, 2.0) 0.2 (0.1, 0.5)
Captain’s support	Overall Aging Young	1.5 (1.0, 2.2) 4.9 (3.1, 7.5) 0.5 (0.3, 0.8)	0.8 (0.4, 1.5) 0.8 (0.3, 2.1) 0.2 (0.1, 0.5)
No age discrimination	Overall Aging Young	1.5 (0.9, 2.7) 4.8 (3.1, 7.4) 0.5 (0.3, 0.8)	1.3 (0.6, 2.9) 0.8 (0.3, 2.2) 0.3 (0.1, 0.6)
***Work satisfaction***	Overall Aging Young		0.4 (0.2, 0.8) 1.1 (0.4, 3.0) 0.2 (0.1, 0.4)

**^1^** Association of psychosocial conditions with job satisfaction and MSDs for all age groups collectively; **^2^** Relative to the middle-aged group.
